# Dark microglia: A new phenotype predominantly associated with pathological states

**DOI:** 10.1002/glia.22966

**Published:** 2016-02-05

**Authors:** Kanchan Bisht, Kaushik P. Sharma, Cynthia Lecours, Maria Gabriela Sánchez, Hassan El Hajj, Giampaolo Milior, Adrián Olmos‐Alonso, Diego Gómez‐Nicola, Giamal Luheshi, Luc Vallières, Igor Branchi, Laura Maggi, Cristina Limatola, Oleg Butovsky, Marie‐Ève Tremblay

**Affiliations:** ^1^ Axe NeurosciencesCentre De Recherche Du CHU De QuébecQuébecQuébecCanada; ^2^ Department of Physiology and PharmacologyIstituto Pasteur‐Fondazione Cenci Bolognetti, Sapienza University of RomeRomeItaly; ^3^ Centre for Biological SciencesUniversity of SouthamptonSouthamptonUnited Kingdom; ^4^ Douglas Mental Health University InstituteDepartment of PsychiatryMcGill UniversityMontrealQuébecCanada; ^5^ Section of Behavioural NeurosciencesDepartment of Cell Biology and NeurosciencesIstituto Superiore Di SanitàRomeItaly; ^6^ Ann Romney Center for Neurologic Diseases, Brigham and Women's Hospital, Harvard Medical SchoolBostonMassachusetts

**Keywords:** microglia, synapses, stress, aging, neurodegenerative diseases

## Abstract

The past decade has witnessed a revolution in our understanding of microglia. These immune cells were shown to actively remodel neuronal circuits, leading to propose new pathogenic mechanisms. To study microglial implication in the loss of synapses, the best pathological correlate of cognitive decline across chronic stress, aging, and diseases, we recently conducted ultrastructural analyses. Our work uncovered the existence of a new microglial phenotype that is rarely present under steady state conditions, in hippocampus, cerebral cortex, amygdala, and hypothalamus, but becomes abundant during chronic stress, aging, fractalkine signaling deficiency (CX_3_CR1 knockout mice), and Alzheimer's disease pathology (APP‐PS1 mice). Even though these cells display ultrastructural features of microglia, they are strikingly distinct from the other phenotypes described so far at the ultrastructural level. They exhibit several signs of oxidative stress, including a condensed, electron‐dense cytoplasm and nucleoplasm making them as “dark” as mitochondria, accompanied by a pronounced remodeling of their nuclear chromatin. Dark microglia appear to be much more active than the normal microglia, reaching for synaptic clefts, while extensively encircling axon terminals and dendritic spines with their highly ramified and thin processes. They stain for the myeloid cell markers IBA1 and GFP (in CX_3_CR1‐GFP mice), and strongly express CD11b and microglia‐specific 4D4 in their processes encircling synaptic elements, and TREM2 when they associate with amyloid plaques. Overall, these findings suggest that dark microglia, a new phenotype that we identified based on their unique properties, could play a significant role in the pathological remodeling of neuronal circuits, especially at synapses. GLIA 2016;64:826–839

## Introduction

The past decade has witnessed a revolution in our understanding of microglia, especially since their roles in the healthy brain have started to unravel (Tremblay et al., 2011). These cells were shown to actively regulate neuronal development, function, and plasticity, providing further insights into their crucial involvement with diseases (Katsumoto et al., 2014; Prinz and Priller 2014; Salter and Beggs, 2014; Tremblay and Sierra, 2014).

Among the discoveries, ultrastructural analyses revealed that IBA1‐positive microglial processes almost exclusively (∼94%) contact synaptic elements (axon terminals, dendritic spines, astrocytic processes, and synaptic clefts) under nonpathological conditions (Tremblay et al., 2010a). Electron microscopy (EM) also indicated that microglial cell bodies and processes frequently engulf axon terminals and dendritic spines, within the thalamus, cerebral cortex, or hippocampus, during development, adulthood, or aging (Milior et al., in press; Paolicelli et al., 2011; Schafer et al., 2012; Tremblay et al., 2010a). With these and other recent studies (Bialas and Stevens, 2013; Elmore et al., 2014; Parkhurst et al., 2013; Rice et al., 2015; Schafer et al., 2012; Stevens et al., 2007), microglia have emerged as crucial effectors of neuronal circuit remodeling in the developing and mature healthy brain (reviewed in Kettenmann et al., 2013; Schafer et al., 2013; Tremblay et al., 2014).

Following up on this work, we are studying microglial implication in the loss of synapses, which is the best pathological correlate of cognitive decline across chronic stress, depression, normal aging, and neurodegenerative conditions that include Alzheimer's disease (AD) (Duman and Aghajanian, 2012; Spires‐Jones and Hyman, 2014). Chronic stress is well known for triggering depression, accelerating aging, predisposing to neurodegenerative diseases, as well as exacerbating their progression and symptoms (Miller and Sadeh, 2014). So far, this work revealed that microglial phagocytosis of synaptic elements is exacerbated in a mouse model of human immunodeficiency virus‐associated cognitive disorder (Lu et al., 2011; Marker et al., 2013; Tremblay et al., 2013) and in wild‐type mice following 2 weeks of chronic unpredictable stress (Milior et al., in press). Microglial phagocytosis of synaptic elements is also elevated in the absence of fractalkine signaling (CX_3_CR1 knockout mice) under basal conditions, but contrarily to wild‐type mice, phagocytosis remains unchanged by chronic stress in these animals, indicating that their experience‐dependent remodeling of neuronal circuits is impaired at synapses (Milior et al., in press).

Working on the latter project and another study regarding microglial involvement in AD using the APP‐PS1 mouse model (Audoy‐Remus et al., 2015), we recently uncovered the existence of a new myeloid cell phenotype, strikingly distinct from the other ones described so far at the ultrastructural level (referred here as “normal” microglia) (Graeber et al., 1988; Herndon, 1964; Mori and Leblond, 1969; Trapp et al., 2007; Tremblay et al., 2010a, 2012 among others). Contrarily to the normal microglia, these cells exhibit several signs of oxidative stress, making them as “dark” as mitochondria. Dark microglia are rarely present under steady state conditions, within the hippocampus, cerebral cortex, amygdala, and hypothalamus, but become abundant upon chronic stress, normal aging, fractalkine signaling deficiency (CX_3_CR1 knockout mice), and AD pathology (APP‐PS1 mice). Dark microglia appear to be phagocytically active, even more than the normal microglia, extensively engulfing dendritic spines, axon terminals, and entire synapses, suggesting their implication in the pathological remodeling of neuronal circuits.

## Materials and Methods

### Animals

All experiments were approved and performed under the guidelines of the Institutional animal ethics committees, in conformity with the European Directive 2010/63/EU and Italian D.lg. 4.05.2014, n. 26 (chronic unpredictable stress), the U.K. Home Office licensing (CCR2 knockout mice), and the Canadian Council on Animal Care guidelines (all the other animals). The animals were housed under a 12‐h light–dark cycle at 22°C–25°C with free access to food and water.

Chronic unpredictable stress experiments were conducted using 12–16‐weeks‐old mice: wild‐type C57BL/6J and CX_3_CR1‐GFP homozygotes on a C57BL/6J background (Jackson Laboratory) where the *Cx3cr1* gene is replaced by a GFP reporter gene (Jung et al., 2000). The animals were housed in Intellicages® (TSE‐system, NewBehavior AG, Zürich, Switzerland). Following 2 weeks of habituation, they were exposed to control (without disturbance) or stressful conditions (chronic unpredictable stress) for two additional weeks, as recently described (Milior et al., in press). The stressful procedures included sporadic air puffs and random modifications of the access to the drinking water. Also, the escape box was removed in the stressful condition, adding on to the stress due to forced social interactions.

For repeated social defeat stress, 7–8‐weeks‐old C57BL/6J mice (Charles River) were subjected to chronic social stress as previously described (Golden et al., 2011). Briefly, the animals interacted 3–5 min daily with CD‐1 retired breeders (4–6 months old) for 10 consecutive days. They were tested on the following day for social interactions with a novel aggressor to evaluate their phenotype of susceptibility to stress, characterized by the avoidance of social interactions. The animals were weighed every third day, and their health status carefully monitored throughout the paradigm. Control C57BL/6 mice were paired‐housed in defeat boxes.

To determine the consequences of amyloid β deposition and aging, we examined 6‐, 14‐, and 21‐month‐old APP‐PS1 mice (Borchelt et al., 1997) as well as 14‐month‐old wild‐type littermate controls. These 6C3‐Tg(APP695)3Dbo Tg(PSEN1)5Dbo/J mice express a chimeric amyloid precursor protein (APPSwe) and the human presenilin 1 (A246E variant) under the mouse prion protein promoter. CCR2 knockout mice (16 weeks old) were used to determine whether the dark microglia could arise from circulating monocytes. CCR2 regulates the egress of monocytes from the bone marrow, resulting in fewer circulating monocytes in CCR2‐deficient mice (Serbina and Pamer, 2006; Tsou et al., 2007). CCR2 knockout mice have a greatly decreased Ly6C^hi^CCR2^+^ monocyte population, and their recruitment to the brain parenchyma is CCR2‐dependent, making them a valuable model to study the role of recruited monocytes in brain function (Gomez‐Nicola et al., 2014; Mildner et al., 2007).

### Tissue Preparation

Three or four mice per group were anesthetized with sodium pentobarbital (80 mg/kg, intraperitoneally) and perfused with 0.1% glutaraldehyde in 4% paraformaldehyde (Ligorio et al., 2009) (chronic unpredictable stress, CCR2 knockout mice) or 3.5% acrolein followed by 4% paraformaldehyde (Tremblay et al., 2010b) (repeated social defeat mice, APP‐PS1 mice, wild‐type controls). Fifty‐micrometer‐thick transverse sections of the brain were cut in sodium phosphate buffer (PBS; 50 mM at pH 7.4) using a vibratome (Leica VT100S) and stored at −20^°^C in cryoprotectant until further processing (Tremblay et al., 2010b). Brain sections containing the ventral hippocampus CA1 (Bregma −3.27 and −4.03 in the stereotaxic atlas of Paxinos and Franklin (2013)), the frontal cortex (Bregma 2.93–2.57), the basolateral nucleus of the amygdala (Bregma −0.83 to −1.55), or the median eminence of the hypothalamus (Bregma −2.03 to −2.27) were examined.

### Immunoperoxidase Staining

Brain sections containing the hippocampus CA1 from the chronic unpredictable stress or repeated social defeat animals were utilized for immunostaining, except for TREM2 staining, which was conducted in 21‐month‐old APP‐PS1 mice. The sections were washed in PBS, quenched, and processed for immunostaining with antibodies against specific cellular and phenotypic markers: ALDH1L1 (Abcam, #ab87117), OLIG2 (Millipore, #AB9610), IBA1 (Wako, #019‐19741), GFP (Aves Lab, GFP‐1020), CD11b (AbD Serotec, MCA711GT), P2RY12, 4C12, and 4D4 (from Oleg Butovsky, Harvard Medical School), MHCII (Millipore, MABF33), TREM2 (Lifespan Biosciences, LS‐C150262), CD11c (BD, clone HL3, 550283), and CD206 (AbD Serotec, MCA2235GA). Secondary antibodies conjugated to biotin were used, all from Jackson ImmunoResearch: goat anti‐rabbit (111‐066‐046), goat anti‐chicken (103‐065‐155), goat anti‐rat (112‐065‐167), and donkey anti‐sheep (713‐066‐147). Briefly, the sections were blocked and incubated overnight at 4^°^C in primary antibody solution, following which they were incubated with appropriate secondary antibody, and then with either ABC Vectastain system (1:100 in Tris‐buffered saline (TBS); Vector Laboratories, #PK‐6100) or Streptavidin‐HRP (Jackson, 016‐030‐084). The sections were developed with diaminobenzidine (0.05%) and hydrogen peroxide (0.015%) to reveal the immunostaining. PBS or TBS was used to prepare the different incubation solutions and also for washing off the excess reagents after the incubation steps. See Table 1 for detailed staining conditions pertaining to each antibody.

**Table 1:** Immunostaining Conditions

Antibody	Animal/condition	Antigen retrieval	Quenching	Blocking	Primary	Secondary	Developing
ALDH1L1	Stressed CX_3_CR1 knockout	–	0.3% H_2_O_2_ in PBS, then 0.1% NaBH_4_ in PBS	5% NGS + 0.5% gelatin in PBS	1:1,000 in blocking buffer	1:500 goat anti‐rabbit in blocking buffer	Incubation with Streptavidin‐HRP made in blocking, 1:1,000
OLIG2	Stressed CX_3_CR1 knockout	–	0.1% NaBH_4_ in PBS, also 0.3% H_2_O_2_ in PBS after secondary	10% FCS + 3% BSA in TBS	1:1,200 in blocking buffer	1:200 goat anti‐rabbit in TBS	Incubation with TBS ABC, 1:100
IBA1	Stressed CX_3_CR1 knockout	–	0.3% H_2_O_2_ in PBS, then 0.1% NaBH_4_ in PBS	10% FCS + 3% BSA, 0.01% Triton in TBS	1:1,000 in blocking buffer	1:300 goat anti‐rabbit in 0.01% Triton TBS	Incubation with 0.01% Triton in TBS ABC, 1:100
GFP	Stressed CX_3_CR1 knockout	–	0.1% NaBH_4_ in PBS, also 0.3% H_2_O_2_ in PBS after secondary	10% FCS + 3% BSA in TBS	1:5,000 in blocking buffer	1:200 goat anti‐chicken in 0.01% Triton TBS	Incubation with TBS ABC, 1:100
CD11b	Stressed CX_3_CR1 knockout	–	0.3% H_2_O_2_ in PBS, then 0.1% NaBH_4_ in PBS	5% NGS + 0.5% gelatin in PBS	1:800 in blocking buffer	1:200 goat anti‐rat in blocking buffer	Incubation with Streptavidin‐HRP made in blocking, 1:1,000
P2RY12	Stressed CX_3_CR1 knockout	–	0.1% NaBH_4_ in PBS also 0.3% H_2_O_2_ in PBS after secondary	10% FCS + 3% BSA in TBS	1:500 in blocking buffer	1:200 goat anti‐rabbit in blocking buffer	Incubation with TBS ABC, 1:100
4C12	Susceptible animals from social defeat stress	–	0.3% H_2_O_2_ in PBS, then 0.1% NaBH_4_ in PBS	5% NGS + 0.5% gelatin in 0.05% Triton PBS	1:50 in blocking buffer	1:200 goat anti‐rat in blocking buffer	Incubation with Streptavidin‐HRP made in blocking, 1:1,000
4D4	Control animals from social defeat stress	Citrate buffer @ 70°C for 40 min	0.3% H_2_O_2_ in PBS, then 0.1% NaBH_4_ in PBS	10% FCS + 3% BSA in TBS	1:1,200 in blocking buffer	1:300 goat anti‐rat in blocking buffer	Incubation with TBS ABC, 1:100
MHCII	Stressed CX_3_CR1 knockout	–	0.3% H_2_O_2_ in PBS after secondary	10% FCS + 3% BSA in TBS	1:500 in blocking buffer	1:200 goat anti‐rat in blocking buffer	Incubation with TBS ABC, 1:100
TREM2	APP‐PS1 21‐months old	Citrate buffer @ 70°C for 40 min	0.3% H_2_O_2_ in PBS, then 0.1% NaBH_4_ in PBS	5% NDS + 0.5% gelatin in PBS	1:100 in blocking buffer	1:300 donkey anti‐sheep in blocking buffer	Incubation with Streptavidin‐HRP made in blocking, 1:1,000
CD206	Susceptible animals from social defeat stress	Citrate buffer @ 70°C for 40 min	0.3% H_2_O_2_ in PBS, then 0.1% NaBH_4_ in PBS	5% NGS + 0.5% gelatin in PBS	1:500 in blocking buffer	1:300 goat anti‐rat in blocking buffer	Incubation with Streptavidin‐HRP made in blocking, 1:1,000
CD11c	Susceptible animals from social defeat stress	Citrate buffer @ 70°C for 40 min	0.3% H_2_O_2_ in PBS, then 0.1% NaBH_4_ in PBS	5% NGS + 0.5% gelatin in PBS + 0.01% Triton	1:500 in blocking buffer	1:300 goat anti‐Armenian hamster in blocking buffer	Incubation with Streptavidin‐HRP made in blocking, 1:1,000

### Light Microscopy

Cellular specificity of each immunostaining was determined at the light microscopic level, using a Zeiss AxioPlan microscope. The immunostained sections were carefully mounted onto glass slides, dehydrated in ascending concentrations of ethanol, cleared in citrisol, and coverslipped with DPX (Electron Microscopy Sciences; EMS).

### Electron Microscopy

Sections stored in cryoprotectant were rinsed in PBS only (ultrastructural and densitometry analyses) or immunostained as described earlier. Afterward, they were postfixed flat in 1% osmium tetroxide and dehydrated in ascending concentrations of ethanol. They were treated with propylene oxide and then impregnated in Durcupan resin (EMS) overnight at room temperature. After mounting between ACLAR embedding films (EMS), they were cured at 55^°^C for 72 h. Areas of interest were excised from the embedding films, re‐embedded at the tip of resin blocks, and cut at 65–80 nm of thickness using an ultramicrotome (Leica Ultracut UC7). Ultrathin sections were collected on bare square mesh grids (EMS), and examined at 80 kV with a FEI Tecnai Spirit G2 transmission electron microscope.

### Qualitative and Quantitative Analyses

Ultrathin sections from the different brain regions and experimental conditions were examined and photographed at various magnifications ranging between 440× and 9,300× using an ORCA‐HR digital camera (10 MP; Hamamatsu). Profiles of neurons, synaptic elements, microglia, astrocytes, oligodendrocytes, and myelinated axons were identified according to well‐established criteria (Peters et al., 1991). In addition to their immunoreactivity for IBA1 or GFP (in the CX_3_CR1‐GFP mice), microglial cells were distinguished from oligodendrocytes by their paler cytoplasm, prevalent association with the extracellular space, distinctive long stretches of endoplasmic reticulum, frequent vacuoles and cellular inclusions, irregular contours with obtuse angles, and small elongated nucleus delineated by a narrow nuclear cistern (Milior et al., in press; Tremblay et al., 2010a). To assess colocalization of the dark microglia with various markers, ultrastructural observations were conducted at the tissue–resin border, where the penetration of antibodies and staining intensity is maximal (Tremblay et al., 2010b). This analysis was strictly conducted in tissue areas where intense immunostaining was observed. For cases where no colocalization was detected, the presence of immunostaining in the same field of view ruled out the possibility that these cells were not stained due to a limited penetration of the antibodies.

To analyze dark microglia's and “normal” microglia's density across control conditions, chronic stress, aging, fractalkine signaling deficiency, and AD pathology, one ultrathin section containing the hippocampus CA1 strata radiatum and lacunosum‐moleculare was sampled in each of three mice per group (3‐month C57Bl/6J control, 14‐month C57Bl/6J control, 3‐month CX_3_CR1 knockout, 3‐month stressed C57Bl/6J, 3‐month stressed CX_3_CR1 knockout, and 14‐month APP‐PS1 model), for a total neuropil surface of ∼400,000 μm^2^ sampled in each animal. The entire section area was sequentially imaged at lowest magnification under the transmission electron microscope (440×) to determine systematically the total number of grid squares enclosing tissue from each of stratum radiatum and lacunosum‐moleculare. These two neuropil layers were identified based on their position to the CA1 pyramidal cell layer, as well as their cellular and subcellular contents. The total surface area was calculated at high precision by multiplying the number of grid squares containing each of stratum radiatum or lacunosum‐moleculare by the area of a single grid square. A schematic representation of all the grid squares included in the analysis was drawn for each section/animal. The ultrathin sections were afterward rigorously screened for the presence of dark microglia, strictly identified based on a series of ultrastructural features that are described in detail in the Results section. Only dark microglia showing a complete or a partial profile where part of the nucleus could be seen were included in the analysis, considering that the chromatin pattern is a distinctive feature of the dark microglia. Each dark microglia was photographed at magnifications between 4,600× and 9,300×, and marked on the schematic representation, for a total of 95 cells included in the analysis. Considering the heterogeneity in dark microglia's distribution, with these cells generally appearing within clusters, and the impossibility to identify them with light microscopy (see Discussion section), and hence to select the areas to examine based on their presence, their density was expressed as maximal numbers per mm^2^ of tissue surface across three animals/experimental conditions. The density of normal microglia was assessed in the same manner to allow for comparison. We did not attempt to distinguish normal microglia from bone marrow‐derived macrophages and other types of myeloid cells in the brain. In addition, using the same samples, we determined the percentage of dark microglia that were: (1) located in stratum radiatum versus lacunosum‐moleculare, (2) directly apposing one or more blood vessel, and (3) encircling one or more synaptic element (axon terminal, dendritic spine, and excitatory synapse between axon terminal and dendritic spine) with their processes.

## Results

### Distinctive Features

**Figure 1:**
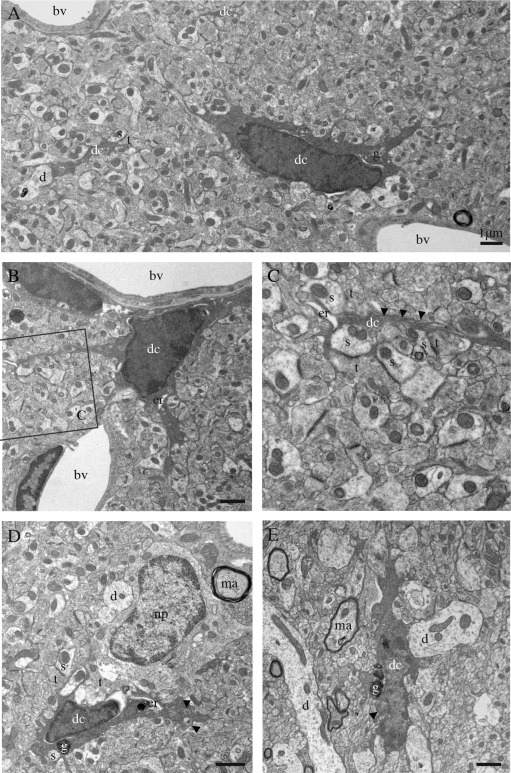
Ultrastructural features of the dark microglia. **A**–**E**: Examples of dark microglial cells (dc) encountered in the CA1 region of the hippocampus (stratum lacunosum‐moleculare) of stressed CX_3_CR1 knockout mice (A–D) or in the median eminence of the hypothalamus in a nontransgenic control mouse (E). In addition to their ultrastructural features of microglia, for instance their frequent long stretches of endoplasmic reticulum (arrowheads in C), these cells are recognized by their various signs of oxidative stress: their condensed, electron‐dense cytoplasm and nucleoplasm, accompanied by cytoplasmic shrinkage, Golgi apparatus (g) and endoplasmic reticulum (er) dilation, and mitochondrial alteration (arrowheads in D, E). Examples of endoplasmic reticulum dilation in cell bodies and a process are, respectively, provided in (B), (D), and (C). The dark microglia contain lipofuscin granules (g) in (D) and (E). Direct contacts with blood vessels (bv), dendrites (d), a neuronal perikaryon (np), axon terminals (t) and dendritic spines (s), and synapses between axon terminals and dendritic spines are also shown. ma = myelinated axon. Scale bars = 1 μm.

**Figure 2:**
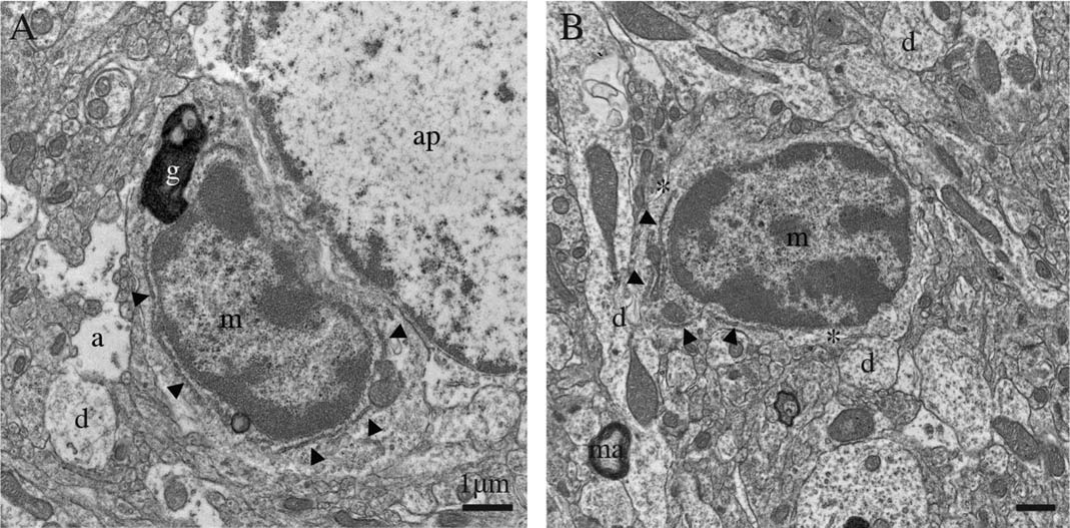
Examples of normal microglia, observed in the median eminence of the hypothalamus in a nontransgenic control mouse. **A**, **B**: Unstained microglia (m) generally display a lighter cytoplasm and nucleoplasm with a clearly defined chromatin pattern, compared with the dark microglial cells. They also share with the dark microglia a small elongated nucleus delineated by a narrow nuclear cistern, associated pockets of extracellular space (asterisks), distinctive long stretches of endoplasmic reticulum (arrowheads), frequent endosomes, lipofuscin granules (g), and cellular inclusions. a = astrocytic process, ap = astrocytic perikaryon, d = dendrites, and ma = myelinated axon. Scale bars = 1 μm.

Using high spatial‐resolution EM, we uncovered the presence of highly phagocytic cells within the brain that have not yet been described, within the hippocampus, cerebral cortex, amygdala, and hypothalamus, across various contexts of health and disease described later. Indeed, we found cells with ultrastructural features of microglia, particularly their size, shape, long stretches of endoplasmic reticulum, frequent interactions with neurons and synapses, and association with the extracellular space. These cells invariably displayed signs of oxidative stress, including a condensed, electron‐dense cytoplasm and nucleoplasm (making them as dark as mitochondria), accompanied by cytoplasmic shrinkage, Golgi apparatus and endoplasmic reticulum dilation, as well as mitochondrial alteration (Fig. 1A–E). By comparison, unstained microglia normally exhibit at the ultrastructural level a light cytoplasm and nucleoplasm with a clearly defined chromatin pattern (Fig. 2A, B). The dark microglia's loss of chromatin pattern suggests an active phenotypic transformation, as chromatin remodeling regulates gene expression, in addition to imparting an epigenetic regulatory control over several key biological processes (Lardenoije et al., 2015).

### Interactions with Synapses

**Figure 3:**
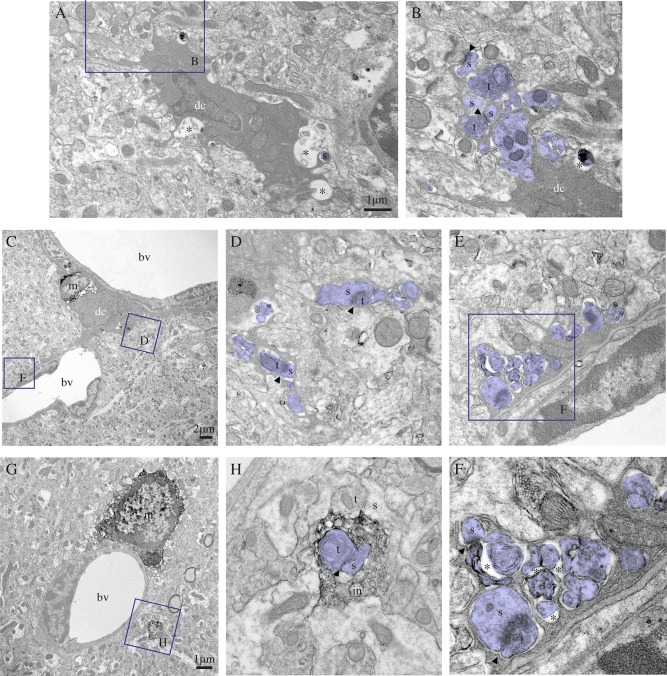
Dark microglia's interactions with synapses. **A**–**F**: Examples of dark microglial cells (dc) typically contacting synaptic elements (colored in purple) with their profusion of highly ramified and extremely thin processes, reaching for synaptic clefts (arrowheads), while encircling axon terminals (t) and dendritic spines (s), in the CA1 lacunosum‐moleculare of stressed CX_3_CR1 knockout mice. In (C), the dark microglia is simultaneously contacting two blood vessels (bv) and a normal microglia (m) that is stained for IBA1. Its processes are extensively encircling various types of synaptic elements, including shrunk axon terminals surrounded by extracellular space (asterisks) in the process of being digested and an entire synapse (see the inset in F). By comparison, an example of IBA1‐stained microglia (m) that is extending a single process, discontinuous from its cell body in ultrathin section, is shown in (**G**). Contrary to the dark microglia processes, it is bulkier and showing obtuse (instead of acute) angles. It nevertheless contains several phagocytic inclusions (in), among which a synapse between an axon terminal (t) and a dendritic spine (s), in addition to making focal contacts (instead of encircling) synaptic elements. Scale bars = 1 μm for (A) and (G) and 2 μm for (C). [Color figure can be viewed in the online issue, which is available at wileyonlinelibrary.com.]

**Figure 4:**
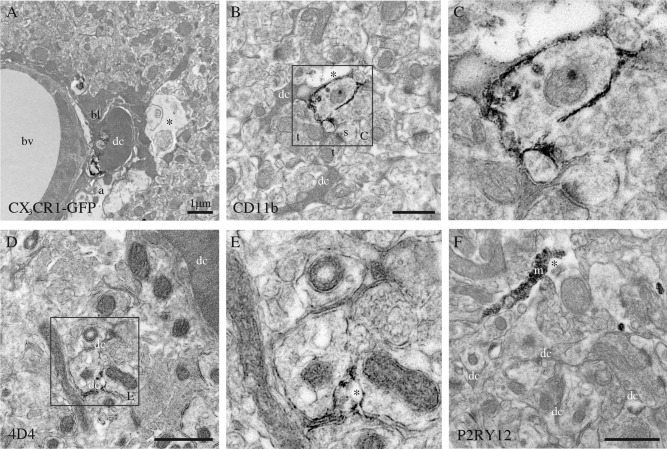
Phenotypic characterization of the dark microglia, using immunoperoxidase staining in the CA1 lacunosum‐moleculare of stressed CX_3_CR1 knockout mice (**A**–**C**, **F**), or a nontransgenic control mouse (**D**, **E**). A: Focal staining for GFP in a dark microglial cell (dc) from a CX_3_CR1‐GFP mouse. In contrast, normal microglia display strong and diffuse immunoreactivity for IBA1 throughout their cytoplasm. B, C: Examples of dark microglia staining for the myeloid cell marker CD11b, which forms CR3 involved in phagocytosis, strongly expressed at the plasma membrane of their processes encircling synaptic elements. D, E: Dark microglia's staining for 4D4, a recently discovered marker of homeostatic microglia, at the extremity of their ramified processes. In contrast, the dark microglia do not stain for P2RY12 (F), another marker of homeostatic microglia that is abundant in microglial processes (m). a = astrocytic process, bl = basal lamina, bv = blood vessel, s = dendritic spine, and t = axon terminals. Asterisks show the extracellular space. Scale bars = 1 μm.

The dark microglia appeared to be extremely active, even more than the normal microglia under both pathological and nonpathological conditions. Dark microglia's processes of primary, secondary, tertiary, and higher‐order, generally contiguous in ultrathin section, often reached for synaptic clefts, suggesting synaptic stripping, and extensively encircled pre‐synaptic axon terminals, postsynaptic dendritic branches and spines, as well as entire synapses between axon terminals and dendritic spines (Fig. 3A–F). Dark microglia's processes typically formed acute angles as they weaved between the other elements of neuropil (Fig. 3B, D–F), akin to astrocytes (Ventura and Harris, 1999), and they were occasionally surrounded by pockets of extracellular space (Figs. 3A and 4A for examples). Together with the shrunken appearance of the encircled synaptic elements (Fig. 3F), these pockets suggest ongoing extracellular digestion in the vicinity of their processes. By comparison, the primary processes of normal microglia rarely protrude from their cell body in ultrathin section (see Fig. 3G for a typical example). Even though higher‐order processes with spindly appearances are encountered (Tremblay et al., 2010a), normal microglia in healthy adult mice generally display bulkier processes with obtuse angles, which contain phagocytic inclusions, and focally contact (rather than encircle) synaptic elements (Fig. 3H).

### Identification

To determine the nature of the dark microglia, we performed pre‐embedding immunostaining to analyze their colocalization with several markers (see Table 1 for details on the immunostaining conditions and Table 2 for a summary of the results). Our results revealed that dark microglia do not express the pan‐astrocytic marker ALDH1L1 or the oligodendrocytic lineage marker OLIG2 (not shown). They displayed a faint and punctiform staining for IBA1 in nontransgenic animals (not shown) or GFP in CX_3_CR1‐GFP mice (Fig. 4A), contrary to the normal microglia that show strong and diffuse immunoreactivity for IBA1 (Fig. 3G, H) and GFP (not shown) throughout their cytoplasm, across contexts of health and disease. Whether this reduction of staining intensity in the dark microglia is due to the condensed state of their cytoplasmic contents, including the GFP and IBA1 proteins, is unclear. However, dark microglia strongly expressed the myeloid cell marker CD11b, which is a critical component of phagocytic receptor CR3, at the plasma membrane of their processes encircling synaptic elements (Fig. 4B, C). They also expressed 4D4, a recently discovered marker of homeostatic microglia (Butovsky et al., 2012), specifically at the extremity of their ramified processes (Fig. 4D, E). In contrast, they were not shown to express P2RY12, another marker of homeostatic microglia (Fig. 4F). Similarly, negative immunostaining results were obtained for markers of dendritic cells (CD11c), perivascular macrophages (CD206) (Galea et al., 2005), and antigen‐presenting cells (MHCII) (not shown).

**Table 2:** Dark Microglia's Phenotypic Characterization in Hippocampus CA1

Immunopositive	IBA1, GFP (in CX3CR1‐GFP mice ), CD11b, 4D4, TREM2
Immunonegative	ALDH1L1, OLIG2, P2RY12, 4C12, MHCII, CD206, CD11c

### Origin

**Figure 5:**
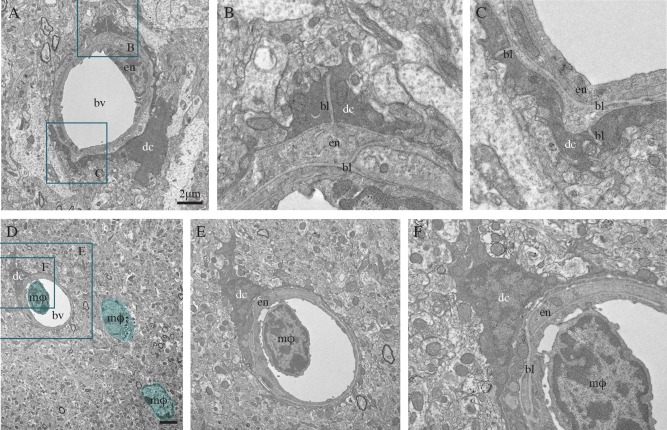
Dark microglia's interactions with the vasculature, in the hypothalamus median eminence of a nontransgenic mouse (**A**–**C**) and CA1 radiatum of an APP‐PS1 6‐month‐old mouse (**D**–**F**). In these two examples, the dark microglial cell (dc) bodies are directly juxtaposing the blood vessels' (bv) basal lamina (bl), and their processes are extending all around the vessels, as well as ensheathing the basal lamina. In (D–F), the blood vessel contains a circulating, bone marrow‐derived monocyte or macrophage (mφ; colored in blue). Additional examples of bone marrow‐derived macrophages can also be seen in the brain parenchyma (colored in blue). It can be noted that the dark microglial cell's contact with the blood vessel is occurring at the site of docking, raising the intriguing possibility of dark cells functional interactions with endothelial cells, as well as circulating immune cells. Scale bars = 2 μm. [Color figure can be viewed in the online issue, which is available at wileyonlinelibrary.com.]

The dark microglia appeared very different from the circulating monocytes that we observed in brain blood vessels and parenchyma (Fig. 5D–F). Given the evidence that Ly6C‐positive monocytes infiltrate the brain in a CCR2‐dependent manner (Mildner et al., 2009), we also examined CCR2 knockout mice in an attempt to determine the origin of the dark microglia. Analysis of these CCR2 knockout mice under steady state conditions revealed examples of dark microglia (not shown), indicating that these cells are either microglia derived from yolk sac or brain progenitors, or bone marrow‐derived cells recruited to the brain in a CCR2‐independent manner (Bruttger et al., 2015), in order to increase the phagocytic capacities. In support of a central origin, the dark cells did not express 4C12 (not shown), a marker of inflammatory monocytes (Butovsky et al., 2014).

### Local and Regional Distribution

Dark microglia were generally found within clusters, and they frequently (65.3% of 95 dark microglia included in the analysis) associated with the vasculature, extending processes all around the blood vessels and ensheathing their basal lamina (Figs. 1B, 3C, 4A, and 5A–D). Even though there was heterogeneity between animals, especially considering the dark microglia's clustered distribution and partial visualization of some of their profiles under the electron microscope, 52.63% were found to be in direct contact with one blood vessel, and 12.63% contacted two vessels simultaneously. Because this analysis was conducted in ultrathin sections, some cells devoid of vascular contact could have apposed blood vessels in another focal plane (i.e., below or above the imaged section), leading to underestimation. We observed dark microglia's interactions with both capillaries and arterioles that we distinguished based on their ultrastructural features.

In the CA1 region of the hippocampus, clusters of dark microglia were mainly found in stratum lacunosum‐moleculare (86.32% of 95 dark cells analyzed in 18 animals), where larger vessels are located, but also to a lesser extent in the radiatum (13.68%; see also Table 3 for densitometry analysis). These two layers contain the apical dendrites of the CA1 pyramidal cells: their distal branches in lacunosum‐moleculare and proximal branches in the radiatum. The CA1 mediates mood and memory, and is profoundly affected by stress (Joels and Krugers, 2007). In the cerebral cortex, dark microglia were encountered in the subgranular layers where larger vessels are also prevalent (not shown). In the amygdala, an integrative center for emotions and motivation, they were observed in the basolateral nucleus (not shown) involved in stress resilience (Berube et al., 2014; Janak and Tye, 2015). In the hypothalamus, dark microglia were identified in the median eminence (Figs. 1E and 5A–C) that contains fenestrated vessels forming a porous blood–brain barrier, and is involved in the hypothalamic‐adenohypophysial regulation of reproduction, stress, lactation, growth, as well as thyroid and metabolic functions (Yin and Gore, 2010).

**Table 3:** Dark Microglia's and Normal Microglia's Layer‐ and Group‐Specific Density in Hippocampus CA1 (cells/mm^2^)

	Dark microglia		Normal microglia		Total	
	Str. radiatum	Str. lacunosum‐moleculare	Str. radiatum	Str. lacunosum‐moleculare	Str. radiatum	Str. lacunosum‐moleculare
3 months						
Wild‐type						
Control	0	0–3.10	7.81–16.25	7.81–37.19	7.81–16.25	7.81–40.29
Stress	0–5.58	2.08–11.16	4.28–10.10	29.93–43.78	4.28–15.58	32.01–54.94
CX_3_CR1 knockout						
Control	0–5.21	0–22.58	3.64–15.63	25.51–34.74	3.64–20.84	25.51–57.32
Stress	0–11.45	6.87–23.08	3.30–13.23	24.26–43.74	3.30–24.68	31.13–66.82
14 months						
Wild‐type—Control	0–2.88	8.15–10.67	8.65–19.02	37.47–55.47	8.65–21.90	45.62–66.14
APP‐PS1—Control	0–2.34	4.67–36.05	5.09–33.27	22.89–52.68	5.09–35.61	27.56–88.73

### Regulation by Stress and Fractalkine Signaling Deficiency

Quantitative analysis of the dark microglia's density in the hippocampus CA1 (strata radiatum and lacunosum‐moleculare) revealed a robust increase in their numbers upon chronic stress and fractalkine signaling deficiency. Their density was found to be maximal in the CX_3_CR1 knockout mice (22.58 cells/mm^2^ in three control CX_3_CR1 knockouts and 23.08 cells/mm^2^ in three stressed CX_3_CR1 knockouts; Table 3), corresponding to approximately half of the normal microglial density (Table 3). Fractalkine signaling deficiency was recently shown to affect microglial migration, survival, dynamic surveillance of the brain, and phagocytic activity toward synaptic elements, with various consequences on the brain and behavior (Arnoux and Audinat, 2015; Blank and Prinz, 2013; Milior et al., in press; Paolicelli et al., 2014). By contrast, we encountered few dark microglia in 3‐month‐old wild‐type controls, showing a maximal density of 3.10 cells/mm^2^ across three animals (Table 3).

### Regulation by Aging and AD Pathology

**Figure 6:**
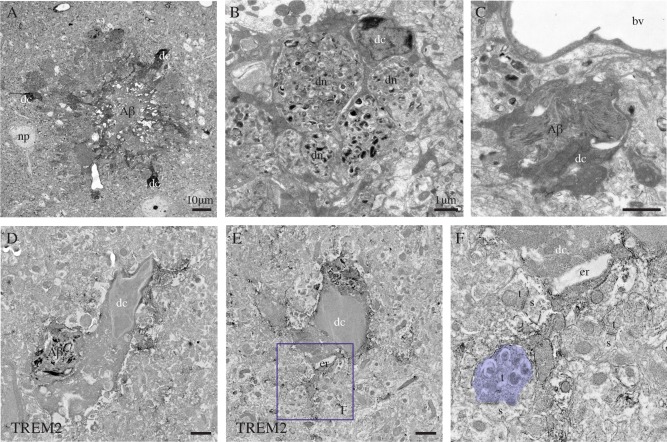
Dark microglia's association with the plaques of amyloid β, examples from the prefrontal cortex subgranular layers (**A**–**C**) and CA1 lacunosum‐moleculare (**D**–**F**) of 6‐ and 21‐month‐old APP‐PS1 mice, respectively. In (A), three dark microglial cells (dc) showing several signs of cellular stress (i.e., extreme condensation, darkening of their cytoplasm and nucleoplasm) are observed in the close proximity of a plaque of amyloid β (Αβ), which is identified by its ultrastructural features. In (B), farther from the plaque, a dark microglial cell process is encircling several dystrophic neurites (dn) containing autophagic vacuoles. In (C) and (D), a dark cell body (D) and process (C) are containing Αβ deposits. D–F: Examples of dark microglial cells that exhibit immunostaining for TREM2, as frequently observed nearby the plaques of Αβ. The remodeling of their nuclear contents can be noted in D and E, together with the pronounced dilation of their endoplasmic reticulum (er) in (E). In addition, the dark microglial cell in (E) is contacting an axon terminal (colored in purple, t) with an accumulation of autophagic vacuoles that makes a synapse on a healthy looking dendritic spine (s). bv = blood vessel and np = neuronal perikaryon. Scale bars = 10 μm for (A), and 1 μm for (B–E). [Color figure can be viewed in the online issue, which is available at wileyonlinelibrary.com.]

Dark microglia became more prevalent during aging, at 14 months of age which corresponds to middle age in non transgenic mice (maximal density of 11.53 cells/mm^2^ in C57BL6/J controls; Table 3), and even more prevalent in aged‐matched APP‐PS1 littermate mice (maximal density of 36.05 cells/mm^2^; Table 3). Nearby the amyloid β plaques (Fig. 6A), dark microglia showing extreme signs of oxidative stress frequently encircled dystrophic neurites (Fig. 6B), and contained amyloid deposits recognized by their ultrastructural features (Fig. 6C, D). Plaque‐associated dark cells typically encircled synaptic elements with signs of dystrophy, such as autophagic vacuoles (Fig. 6F) as previously described in AD models (Francois et al., 2014; Sanchez‐Varo et al., 2012). Most of these dark microglia were TREM2 immunoreactive (Fig. 6D–F), suggesting that they could be the plaque‐associated TREM2‐positive myeloid cells which—regardless of their still debated origin (Rivest, 2015)—were recently shown to express the phagocytic effectors MERTK and AXL (Jay et al., 2015; Savage et al., 2015).

## Discussion

Our work describes a novel microglial phenotype with ultrastructural features that imply unique properties as compared with the other myeloid cell phenotypes that we encountered in the brain. The dark microglia that we described are predominantly associated with pathological states. They are identified by the condensation of their cytoplasm and nucleoplasm (making them look “dark” with EM), accompanied by various alterations, such as endoplasmic reticulum dilation, which is the most well‐characterized sign of oxidative stress at the ultrastructural level (Schonthal, 2012). They appear to be extremely active, typically engulfing dendritic spines and axon terminals, and at times entire synapses. Their nuclear chromatin remodeling suggests changes at the transcriptional and epigenetic levels that are indicative of ongoing DNA replication and repair, apoptosis, chromosome segregation, or a state of pluripotency (Lardenoije et al., 2015).

To our knowledge, the only other mention of dark microglia within the literature comes from a pioneering EM study published over 50 years ago that examined microglia in the rat parietal cortex (Schultz et al., 1957). Without mentioning dark microglia specifically, this publication described microglia as cells with a “striking overall electron density”, making it impossible to delineate their nucleus. Their processes displayed “heavy material concentration” and based on the pictures provided, they also appeared highly ramified and phagocytic, as for the dark microglia. Additionally, these “microglia” showed a uniform chromatin pattern, contrary to the normal microglia, which were subsequently described using staining with del Rio‐Hortega's silver carbonate method (Mori and Leblond, 1969) and more contemporarily immunostaining for IBA1 (Shapiro et al., 2009; Tremblay et al., 2010a).

It is fundamental at this stage to identify the nature and origin of the dark microglia, especially considering their extreme phagocytic activity at the synapse, and high potential for therapeutic intervention. The dark microglia could represent a newly discovered myeloid cell that infiltrates the brain in a CCR2‐independent manner, considering their occurrence in the CCR2 knockout mice, which would be recruited to increase the brain's phagocytic capacities in contexts where the pathological or traumatic remodeling of neuronal circuits is exacerbated. They do not express 4C12, a marker of inflammatory monocytes (Butovsky et al., 2014). Nevertheless, they could arise from the infiltration and subsequent differentiation of bone marrow‐derived cells into the brain (Bruttger et al., 2015). Alternatively, dark microglia could represent a subset of hyperactive microglia that become stressed as a result of their hyperactivity in contexts of adversity, leading to dysregulated interactions with synapses. By comparison, normal microglia were shown to become “senescent” with age and AD pathology (Siskova and Tremblay, 2013), accumulating phagocytic debris, as well as showing slower process motility and response to injury (Hefendehl et al., 2014; Heppner et al., 2015; Tremblay et al., 2012).

Tridimensional serial block‐face EM will be required for a more complete account of the dark microglia's changes in density, morphology, and interactions with neurons, synapses, and the neurovascular unit across various brain regions, and contexts of health and disease. Because dark microglia are currently recognizable only by EM (staining for IBA1, GFP (in the CX_3_CR1‐GFP mice), CD11b, 4D4, or TREM2 is not sufficient to distinguish them from other myeloid cells at the light microscopy level), it will be important to find selective markers enabling to precisely identify them, in order to study their dynamic behavior, gene expression signature, pro‐ or anti‐inflammatory activity, and establish their functional relevance to the pathogenesis of diseases. In parallel, the presence of dark microglia will have to be confirmed in human postmortem brain samples.
